# Multimodal Imaging in the Diagnosis of Primary Aortic Epithelioid Angiosarcoma

**DOI:** 10.7759/cureus.90295

**Published:** 2025-08-17

**Authors:** Kai Wakatsuki, Shigeshi Kohno, Shojiro Oka, Ban Yoshihito, Ryutaro Onishi, Toshiyuki Noda, Kumiko Ando, Tsuyoshi Suga, Shigeo Hara, Shigeki Arizono

**Affiliations:** 1 Diagnostic Radiology, Kobe City Medical Center General Hospital, Kobe, JPN; 2 Diagnostic Radiology, Kitano Hospital, Osaka, JPN; 3 Pathology, Kobe City Medical Center General Hospital, Kobe, JPN

**Keywords:** aortic intimal angiosarcoma, atherothrombotic lesions, cine imaging, fdg-pet/ct, magnetic resonance imaging, multimodal imaging

## Abstract

Primary malignant tumors of the aorta (PMTA) are rare, yet highly aggressive neoplasms associated with poor prognosis. The preoperative diagnosis of these tumors remains challenging, as conventional imaging modalities, including computed tomography (CT) and magnetic resonance imaging (MRI), frequently fail to distinguish PMTA from atherothrombotic lesions. This case highlighted the efficacy of multimodality imaging, particularly F-18 fluorodeoxyglucose-positron emission tomography/computed tomography (FDG-PET/CT), in diagnosing PMTA, such as aortic intimal angiosarcomas.

A 73-year-old male patient presented with a history of intermittent fever and persistently elevated inflammatory markers over a three-month period. Initial CT revealed a pedunculated intraluminal mass within the descending aorta, accompanied by multiple infarctions of the spleen and kidneys. No apparent contrast enhancement of the lesion was observed on CT. MRI demonstrated mildly high-signal intensity on T1- and T2-weighted images, without marked contrast enhancement. Cine MRI indicated tumor margin mobility, suggesting a dynamic lesion, rather than a fixed thrombus. FDG-PET/CT revealed abnormal hypermetabolic activity within the aortic lesion, with a maximum standardized uptake value of 10.8. Moreover, this modality identified distant metastases within the ribs and femoral head. The patient subsequently underwent descending aortic replacement surgery, and histopathological analysis confirmed the diagnosis of an epithelioid angiosarcoma. Despite postoperative chemotherapy, the patient died due to the progression of brain metastases.

This case confirmed the essential role of FDG-PET/CT in distinguishing PMTA from atherothrombotic lesions. Due to the poor prognosis and aggressive nature of PMTA, early diagnosis through multimodal imaging is paramount in guiding appropriate clinical management and improving treatment outcomes.

## Introduction

The prognosis for primary aortic tumors is extremely poor. The median overall survival following diagnosis is approximately 15.6 months, whereas patients with intimal sarcoma demonstrate a markedly shorter mean survival of 9.8 months [1]. Although diagnosis typically involves a combination of multiple imaging modalities, including computed tomography (CT), magnetic resonance imaging (MRI), and F-18 fluorodeoxyglucose-positron emission tomography (FDG-PET)/CT, no standardized diagnostic approach has been established. Preoperative diagnosis remains challenging, as CT and MRI regularly fail to distinguish these tumors from thrombi [2]. Herein, we present a case of PMTA that was indistinguishable from a thrombus on CT and MRI but was successfully identified using FDG-PET/CT.

## Case presentation

A 73-year-old male patient presented with an intermittent fever and persistently elevated inflammatory markers for a three-month duration. He reported no relevant medical history, including any history of cancer.

On admission, laboratory test results revealed an elevated C-reactive protein level of 8.10 mg/dL, while other tests, including coagulation studies, demonstrated no abnormal findings of clinical significance.

**Figure 1:**
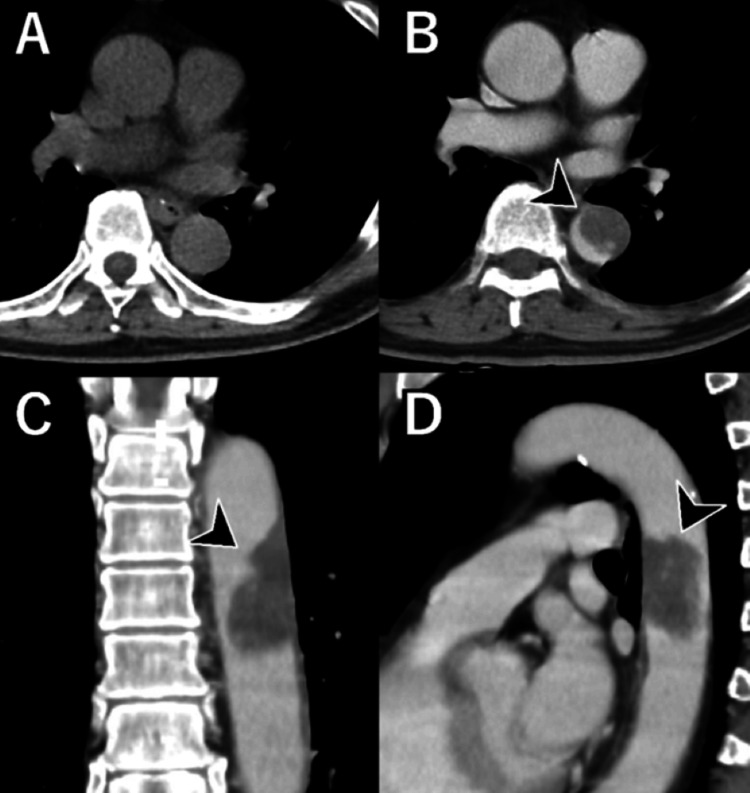
Contrast-enhanced computed tomographic images. On non-contrast CT, the tumor was poorly visualized (A). On contrast-enhanced CT, a 25 mm-diameter, pedunculated tumor protruding into the descending aorta is observed (B, C, D: arrowheads). Contrast enhancement of the lesion was not clearly observed.

Contrast-enhanced CT revealed a 25-mm-diameter, pedunculated mass protruding into the descending aorta (Figure 1), along with multiple infarctions of the spleen and kidneys. No apparent contrast enhancement of the lesion was observed on CT. These findings initially suggested the presence of a mural thrombus.

**Figure 2:**
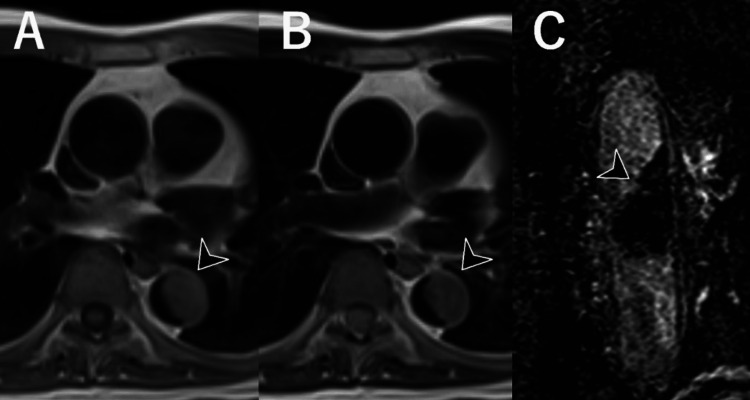
Contrast-enhanced magnetic resonance images. The lesion exhibits mildly high signal intensity on T1-weighted imaging (A). Moreover, a slightly higher signal intensity than that of muscle is noted on T2-weighted imaging (B). No apparent contrast enhancement is observed on subtraction imaging (C).

However, the lesion’s presentation was atypical, with few signs of atherosclerosis in other arterial regions, making this diagnosis less likely. Due to this atypical presentation, an MRI was performed for further characterization. MRI revealed the lesion to have mildly high-signal intensities on both T1- and T2-weighted imaging, with no clear enhancement on subtraction images (Figure 2). The signal intensity appeared relatively homogeneous, and no obvious contrast enhancement was visually evident.

However, cine imaging demonstrated mobility at the lesion margin (Video 1), suggesting the possibility of a dynamic lesion rather than a fixed thrombus.

**Video 1:** Cine magnetic resonance imaging (MRI). Cine MRI demonstrates mobility at the tumor margin.

**Figure 3:**
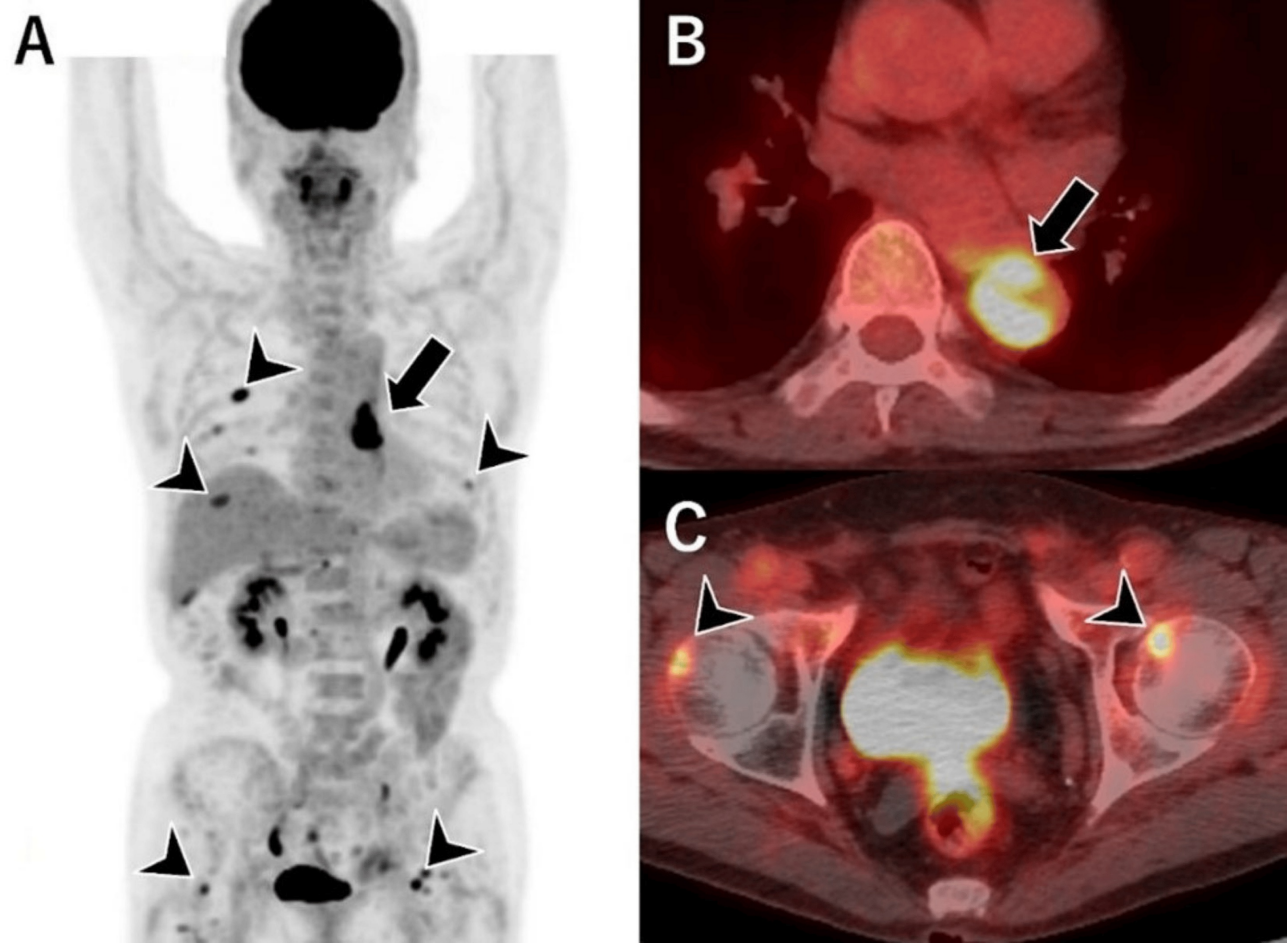
F-18 fluorodeoxyglucose-positron emission tomography (FDG-PET)/CT images. A mass exhibiting abnormal accumulation, with a maximum standardized uptake value of 10.8, is identified in the descending aorta (A, B: arrows), along with additional metastatic lesions observed in the ribs and femoral heads (A, C: arrowheads).

Both CT and MRI failed to reveal definitive findings indicative of malignancy. Nonetheless, due to the paucity of atherosclerotic changes in other vascular regions and the presence of other atypical features, malignancy was considered. FDG-PET/CT was performed for further evaluation. The subsequent FDG-PET/CT showed abnormal hypermetabolic activity within the aortic lesion, with a maximum standardized uptake value of 10.8. Furthermore, additional areas of increased FDG uptake in the ribs and femoral head were detected, consistent with metastatic disease (Figure 3). This metabolic evidence of malignancy was the decisive factor that shifted the clinical diagnosis from a thrombus to a neoplasm, ultimately prompting the decision for surgical intervention. 

**Figure 4:**
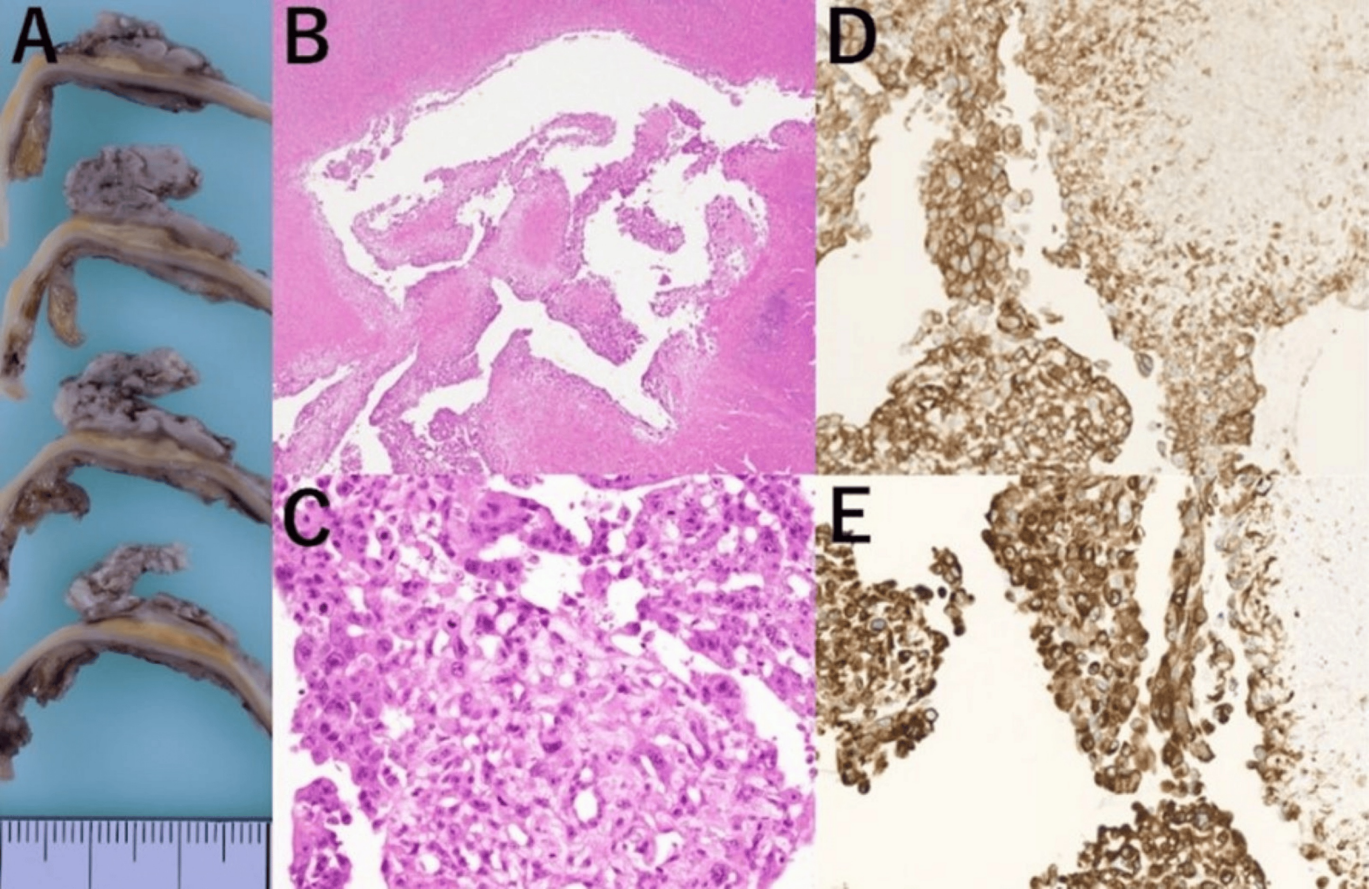
Histopathological findings. Macroscopically, a tumor protrudes into the lumen from the aortic wall (A). Hematoxylin and eosin staining reveals atherosclerotic plaques with cholesterol clefts, continuous with the aortic wall (B, C). Immunohistochemical staining shows predominant positivity for cluster of differentiation 31 (D), a marker of endothelial origin; in addition to cytokeratin (E), a marker of epithelial-like cells. Based on these histopathological findings, the lesion has been diagnosed as a primary aortic epithelioid angiosarcoma.

The patient underwent descending aorta replacement surgery. Histopathological examination revealed atherosclerotic plaques with cholesterol clefts, necrotic debris, and extensive atypical cells on the surface. Immunohistochemistry showed diffuse positivity for cluster of differentiation (CD) 31 and cytokeratin MNF116, partial positivity for CD34, and negativity for alpha-smooth muscle actin, Podoplanin, CD45, and S100 protein (Figure 4). Mindbomb homolog-1 labeling index was approximately 40%. These findings confirmed the diagnosis of primary aortic epithelioid angiosarcoma. A standardized staging system applicable to the primary site of this tumor has not been established; therefore, tumor node metastasis (TNM) or overall stage classification is not provided in this report. Despite chemotherapy, the patient died as a result of brain metastases 4 months postoperatively.

## Discussion

PMTA has a poor prognosis, with a median survival of 11 months and three-year and five-year survival rates of 17.1% and 8.8%, respectively [3]. PMTA frequently manifests as thromboembolic events that often mimic atherothrombotic disease, leading to misdiagnosis [4]. Surgical resection remains the primary treatment when feasible; however, most cases are diagnosed at an advanced stage, limiting therapeutic options [5]. While complete resection combined with radiation therapy provides the best prognosis for localized disease, current knowledge is mainly based on case reports and small case series [5]. Due to the importance of early diagnosis and the frequency of misdiagnoses, comprehending the distinguishing imaging features of PMTA is essential.

The diagnostic approach to PMTA has considerably evolved with advances in imaging technology. Contrast-enhanced CT serves as an initial diagnostic tool. However, mural tumors may be misinterpreted as atherosclerotic lesions [5]. When evaluating differential diagnoses, the possibility of a PMTA should be considered in cases where a cardioembolic source has been excluded with no evidence of widespread aortic atherosclerosis [6]. Specific imaging features suggestive of aortic intimal angiosarcomas include a pedunculated appearance, an atypical location for thrombus formation, extension beyond the vascular wall, and contrast enhancement [5]. CT alone has limited diagnostic utility in differentiating an aortic intimal angiosarcoma from a thromboatherosclerotic lesion. Previous studies have described extension beyond the vascular wall and contrast enhancement as CT features indicative of aortic intimal angiosarcomas. When contrast-enhanced CT reveals an intraluminal mass-like lesion without clear evidence of atherosclerotic changes in other vascular territories, the potential for malignancy should be considered. Subsequently, additional imaging modalities should be employed to facilitate a more accurate and comprehensive diagnosis.

To the best of our knowledge, no studies have specifically reported on the utility of non-contrast MRI signal characteristics in differentiating PMTA from a thromboatherosclerotic lesion. Contrast-enhanced MRI effectively identifies enhancement of the aortic mass, thereby supporting the diagnosis of an angiosarcoma [5]. However, detecting contrast enhancement in PMTA using MRI may not always be straightforward [7]. Therefore, the absence of enhancement should not be regarded as definitive in the exclusion of PMTA, and careful interpretation in conjunction with imaging findings is essential. In the present case, the lesion exhibited relatively homogeneous signal intensity on non-contrast MRI. Furthermore, neither contrast-enhanced CT nor MRI demonstrated notable enhancement, making differentiation from a thromboatherosclerotic lesion more challenging. Although the absence of contrast enhancement on MRI may be attributed to the inability to visually detect subtle enhancement, it may also reflect the histopathological finding that neoplastic cells were distributed along the surface of the atherosclerotic plaque.

In our case, tumor margin mobility observed on cine imaging indicated a dynamically moving lesion rather than a fixed one. Transesophageal echocardiography may assist in discriminating between lesions by assessing lesion mobility [8]; similarly, MRI cine imaging might aid in distinguishing PMTA from thrombi.

Multiple case reports have demonstrated the efficacy of FDG-PET/CT in diagnosing PMTAs, highlighting its role in detecting hypermetabolic activity suggestive of malignancy [6,9]. In the present case, FDG-PET/CT revealed abnormal FDG uptake within the lesion, which played a decisive role in confirming the diagnosis. FDG-PET/CT may have a potential role in diagnosing PMTA, as it demonstrates hypermetabolic activity within the aortic wall and may aid in identifying metastatic disease [7]. A previous report has also indicated that FDG-PET/CT may be useful in differentiating thrombi from malignancies in other vascular territories [10]. Moreover, recent studies have demonstrated the utility of FDG-PET/CT in differentiating pulmonary artery sarcomas from pulmonary thromboembolisms, with pulmonary artery sarcomas showing much higher glucose metabolism [10].

Within the scope of our literature search, no reports were found demonstrating the diagnostic utility of fibroblast activation protein inhibitor (FAPI) PET/CT for aortic sarcoma; however, a case report in pulmonary artery intimal sarcoma, in which FDG PET/CT and 68Ga-FAPI-04 PET/CT were performed in the same patient, showed that 68Ga-FAPI-04 PET/CT provided higher tumor-to-background contrast and more clearly delineated the tumor extent compared with FDG PET/CT, attributable to minimal physiological uptake in the normal pulmonary arterial wall and right ventricular wall [11]. This case may suggest the potential usefulness of FAPI PET/CT in the diagnosis of PMTA.

[177Lu]Lu-FAPI, a novel radioligand targeting FAP abundantly expressed in cancer-associated fibroblasts, has rapidly emerged as a promising theranostic agent for solid tumors with limited treatment options [12]. The current clinical evidence specifically regarding [177Lu]Lu-FAPI remains in its early stages, and further evidence regarding its potential effectiveness for PMTA, as suggested by the present case, is anticipated.

In the present case, no enhancement was observed on contrast-enhanced CT and dynamic contrast-enhanced MRI, complicating the differentiation between PMTA and thrombus. FDG-PET/CT could potentially aid in distinguishing tumors from thrombi by detecting elevated metabolic uptake. However, FDG-PET/CT is typically not performed unless malignancy is suspected. Thus, radiologists should be familiar with the imaging characteristics of these tumors.

## Conclusions

PMTA is rare and frequently misdiagnosed due to its nonspecific presentation and thromboembolic manifestations. CT and MRI may present challenges in differentiating PMTAs from thromboatherosclerotic lesions. However, evaluating lesion morphology, assessing for the presence of atherosclerosis in other vascular regions, and integrating findings from multiple imaging modalities are essential for an accurate diagnosis. Therefore, FDG-PET/CT plays a key role in differentiating PMTA from thromboatherosclerotic lesions by identifying hypermetabolic activity.
